# General Culture for the Dentist

**Published:** 1873-05

**Authors:** S. H. Harlan

**Affiliations:** Mechanicsburg, O.


### GENERAL CULTURE FOR THE DENTIST.

BY S. H. HARLAN, MECHANICSBURG, O.

It is quite unnecessary to say aught touching the indispensable need of careful study and preparation in each and every department of science, out of which the intelligent and successful practice of our specialty can only be evolved.

Certainly that would be superfluous, since it has passed into universal acknowledgment among the better educated members of our profession, that now and hereafter, thorough and patient study of the commonly prescribed text books must at least precede the assumption of the duties and responsibilities of the dentist,—a preparation and training so conscientious and exhaustive, that the censors before whom he must appear for examination shall readily recognize his attainments, and freely accord him professional fellowship.

All this is understood and admitted.

But after that what? Is it enough, will it suffice, for an honorable and successful career, that only the prescribed studies pertaining to his profession be mastered? Have the obligations or necessity for a broader culture been sufficiently urged upon us as members of a liberal, an enlightened and progressive profession? I believe that some of us have grown into the notion that our attainments will be deep and high enough, and sufficiently cyclopedic, when we have absorbed the text books and literature of dentistry merely.

Now, of the some thousands of dentists in our country enjoying a reputable practice, probably not one half the number have had the benefits of an University education.

The fine, sharp, mental discipline, the outgrowth of such opportunities — that drawing out and rounding up of our faculties, incident to such training, is inaccessable to a considerable proportion of the present members of the dental profession.

Many of us, who forty years ago vegetated in rural districts, enjoyed no better facilities for acquiring the then common English education, than what was furnished by the country school. Taking that very moderate and rudimentary stock of lore, as the basis and body of our culture. And if what I offer to-day has bearing and significance for any, it is for precisely that portion of our brethren. And the writer hereof frankly acknowledges himself as one.

What though age, and increased duties, may appear as bars to habits of study, yet much can be done toward adding to that repertory of facts and principles, which really underlie all high and noble achievements. There is much to be accomplished yet for such as we. And while it is all too late to commence a *systematic* study of the classics, and the exact sciences, too late to do fully and *completely* the work that is best, and most lastingly done during the susceptible and even ardent days of youth, yet we need not go barren of a passable and even comprehensive knowledge of the majority of leading sciences outside of, and beyond our specialty.

*We should read.*

Some of us have leisure for much reading, while most of us do read frequently and voluminously ; but alas, too large a percentage of our reading, I fear, is of an ephemeral clap-trap character, represented by the *light* literature of the day ; but most notably by the ever ubiquitous daily newspaper.

Not only should we familiarize ourselves with the best productions of pure literature, but grow largely acquainted with physical sciences as interpreted by their ablest exponents. We can, so to say, put ourselves in rapport with the illustrious phalanx of thinkers and scientists of the present and near past, and be quickened and stimulated by the contact.

It is but to repeat a hackneyed truism to say that we live in a progressive epoch.

In recalling the remarkable discoveries in physical science within a quarter of a century past, made since Humboldt gave to the world his immortal "Cosmos,"—the diseovery of the mechanical equivalent of heat, Spectrum Analysis, Tyndall's wonderful experiments and discoveries, the enunciation of the great naturalist's hypotheses of the Origin of Species, one can not but feel a pardonable pride in believing that he lives and thinks in a period of mental activity unsurpassed if ever paralelled in the career of mind.

You may answer—"what has all this to do with the progress of dentistry?" Well, directly it may have no intimate, no close relationship; yet, indirectly it has much.

For though in Herbert Spencer's "First Principles" is found no hint as to the best materials for artificial dentures, or Huxley's "Lay Sermons no word on Dental Hygiene, and though Lubbock and Lyell remain silent on first dentition or the eruption of the sixth year molars, and so on of Mill, Bain, Carlyle, Hamilton and the rest, yet I hold that by the mere effort to grasp their statements and assimilate their intellectual treasures, we strengthen our mental faculties and fertilize and vivify our powers of reason, observation and analysis:—In short we prepare ourselves to be more industrious, cautious, deductive and philosophic, in every line of inquiry and experiment which the practice of our profession may from time to time evoke.

Let me trust that no member of this society leans toward the opinion that the dentist should essentially and practically be a person of one idea—no matter how intent upon it he may be, no matter if he would consecrate himself to that end. But rather let us recognize as a dominant truth that by widening the scope of our mental perspective, instead of growing superficial and diffuse, we may become scholarly, ripe in judgment, accurate in execution; and thus useful and dignified members of our profession, and cultured society at large.

